# Assessment of Antimicrobial Activity of Nanocomposites Based on Nano-Hydroxyapatite (HAP), Chitosan, and Vitamin K2

**DOI:** 10.7759/cureus.53339

**Published:** 2024-01-31

**Authors:** Revathi Duraisamy, Dhanraj Ganapathy, Rajeshkumar Shanmugam, Ezhilarasan Devaraj, Amrutha Shenoy

**Affiliations:** 1 Department of Prosthodontics, Saveetha Dental College, Saveetha Institute of Medical and Technical Sciences, Saveetha University, Chennai, IND; 2 Department of Pharmacology, Saveetha Dental College, Saveetha Institute of Medical and Technical Sciences, Saveetha University, Chennai, IND; 3 Department of Prosthodontics and Implantology, Saveetha Dental College, Saveetha Institute of Medical and Technical Sciences, Saveetha University, Chennai, IND

**Keywords:** health, dental, biofilm, implant coatings, disk diffusion test, chitosan, hydroxyapatite nanoparticles, vitamin k2, nanocomposites, antimicrobial

## Abstract

Objective: The objective of this study was to evaluate the antimicrobial potential of nanocomposites containing vitamin K2, hydroxyapatite nanoparticles (nHAP), and chitosan (Chito)-coated dental implants against clinically relevant microbial strains.

Materials and Methods: Four test compounds were prepared: vitamin K2 + nHAP, K2 + Chito + nHAP, vitamin K2, and vitamin K2 + Chito. Agar well diffusion test was conducted to assess the antimicrobial activity of these compounds against *Staphylococcus aureus (S. aureus), Streptococcus mutans (S. mutans), Enterococcus faecalis (E. faecalis),* and *Candida albicans (C. albicans).*

Results: The vitamin K2 + nHAP nanocomposite exhibited antimicrobial activity against all tested microorganisms, with *E. faecalis* showing the highest sensitivity (25 mm zone of inhibition at 100 µL concentration). The K2 + Chito + nHAP nanocomposite demonstrated potent antimicrobial activity with *C. albicans* displaying the highest sensitivity (28 mm zone of inhibition at 100 µL concentration). Pure vitamin K2 showed limited antimicrobial activity, vitamin K2 combined with chitosan exhibited significant susceptibility to *C. albicans*, resulting in a substantial inhibition zone of 24 mm diameter at a concentration of 100 µL.

Conclusion: The synergistic effects of vitamin K2 with nHAP and chitosan highlight the potential of these nanocomposites for biomedical applications. These findings contribute to the development of effective nanocomposites to address antimicrobial resistance and improve infection control in various biomedical fields.

## Introduction

In recent decades, the alarming surge in antibiotic-resistant microbial strains has posed a significant threat to public health globally [1,2]. The limitations of conventional antimicrobial agents such as antimicrobial resistance and ineffectivity for some endospores and viruses have necessitated the exploration of novel approaches to combat infections effectively. Nanotechnology has emerged as a promising avenue in this regard, offering innovative solutions for antimicrobial applications [3]. Nanostructured materials have shown remarkable potential in overcoming the challenges posed by drug-resistant microbes, paving the way for the development of effective nanocomposites with enhanced antimicrobial properties [4]. Hydroxyapatite (HAP), a biocompatible and osteoconductive material, has garnered considerable interest in various biomedical devices and targeted drug delivery. Its exceptional chemical stability, nontoxic nature, and excellent biocompatibility render it an ideal candidate for medical applications [5]. HAP is a primary mineral in bone formation and is encased in a collagen triple helix framework, nanocrystals of both pure nHAP and HAP, when integrated with polymers, have been harnessed in drug delivery contexts. The exploration of how their physical and chemical attributes correlate with biological uses has emerged as a highly intriguing research topic [6,7].

Additionally, chitosan, derived from chitin, obtained from natural sources such as shells of shrimp and other sea crustaceans is a biopolymer known for its biodegradability, biocompatibility, and nontoxicity. These attributes make chitosan an attractive matrix for incorporating antimicrobial agents [8,9]. Vitamin K is a member of a collection of fat-soluble substances characterized by a foundational structure of 2-methyl-1,4-naphthoquinone and an isoprenoid chain positioned at the 3-position. These different forms of vitamin K, known as vitamin K1 (phylloquinone), vitamin K2 (menaquinone), and vitamin K3 (menadione), are classified based on the structure of their side chains [10].

Vitamin K2 has emerged as a promising antimicrobial agent, in addition to its traditional roles in blood clotting and bone health. It exhibits inhibitory effects against various bacterial and fungal pathogens as it is believed to disrupt microbial cell membranes and interfere with essential cellular processes [11]. Moreover, vitamin K2's immunomodulatory properties may enhance the body's ability to combat infections by stimulating the production of antimicrobial peptides. Combining vitamin K2 with conventional antimicrobial agents may lead to synergistic effects, offering potential solutions to combat antibiotic resistance [12,13]. Studies have suggested that vitamin K2 prepared from metabolic engineering will likely to possess antimicrobial activity against a wide spectrum of pathogens [13], making it an intriguing candidate for synergistic combination with other antimicrobial agents. This study provides an information on metabolic engineering methods for vitamin K2 production by expanding the product spectrum [13].

The combination of HA, chitosan, and vitamin K2 to form a nanocomposite presents a compelling strategy for developing advanced antimicrobial materials. The synergistic effects of these constituents are anticipated to result in a powerful and effective antimicrobial agent that can address the growing concerns of antibiotic resistance and improve infection control. The rationale behind this research lies in the need for new and potent antimicrobial agents that can efficiently combat infections while minimizing the risk of resistance development. By harnessing the unique properties of nano-hydroxyapatite, chitosan, and vitamin K2, we aim to synthesize a nanocomposite with enhanced antimicrobial activity, paving the way for innovative therapeutic approaches. This study aimed to explore the antimicrobial capabilities of the nanocomposite composed of nano-hydroxyapatite, chitosan, and vitamin K2 against a range of microorganisms with clinical significance.

## Materials and methods

The research protocol was developed to assess the potential of nanocomposites containing vitamin K2, nano-hydroxyapatite (nHAP), and chitosan, either individually or in combination, as novel antimicrobial agents. Before commencing the study, ethical approval was obtained from the Scientific Review Board- Saveetha Dental College (SRB/SDC/PhD/PROSTHO-2105/21/TH-094).

Study type and setting

The cross-sectional study, spanning a period of three months, was conducted from September 2023 to November 2023 at the Department of Microbiology, Saveetha Dental College and Hospital.

Preparation of test solutions

Three primary solutions were purchased and were of analytical grade. The process for preparing each test solution is outlined below, detailing the distinctive steps taken for the synthesis of each compound.

Chitosan Solution

Chitosan solution was prepared by dispersing the chitosan (Sigma-Aldrich Chemie GmbH, Germany) in 2% acetic acid (v/v) for 1 h at room temperature (25°C) with magnetic agitation (430 rpm) stirring until the biopolymer was fully dissolved. Thereby achieving a final concentration of 10 mg/mL of Chitosan in the solution.

Vitamin K2 Solution

150 mg of Vitamin K2 was dispersed in 15 mL of distilled water and was thoroughly mixed to achieve complete dissolution of Vitamin K2 in water, thereby achieving a final concentration of 10 mg/mL of vitamin K2 in the solution.

Nano-Hydroxyapatite Solution

A 2.5 μm hydroxyapatite (HAp) was precipitated with the use of calcium chloride (CaCl2) and disodium hydrogen phosphate (Na2HPO4) (Sigma-Aldrich Chemie GmbH, Germany). It was dispersed in stock distilled water. Thorough mixing was performed, and for precise particle size control, filtration was considered. The nHAP solution was stored in a suitable container under recommended conditions.

Table 1 will give brief information about chemical reagents used and their manufacturer.

**Table 1:** Chemical Reagents and Manufacturer

Chemical reagent	Manufacturer
Vitamin K2 (CAS No: 863-61-6)	Merck, Darmstadt, Germany
Chitosan (CAS No: 9012-76-4)	Sigma-Aldrich Chemie GmbH, Germany
Nano-hydroxyapatite (CAS No: 12167-74-7)	Sigma-Aldrich Chemie GmbH, Germany
Distilled water (CAS No: 7732-18-5)	Merck, Darmstadt, Germany
Mueller Hinton agar	HiMedia, Thane, India
Rose Bengal agar	

From these test solutions, four groups of compounds were prepared for antimicrobial testing: vitamin K2 + nano-hydroxyapatite (VK2 + nHAP); vitamin K2 + chitosan + nano-hydroxyapatite (VK2 + Chito + nHAP); vitamin K2 only (VK2); vitamin K2 + chitosan (VK2 + chitosan).

Preparation of test compounds

The vitamin K2 solution was prepared by dissolving vitamin K2 powder (Merck, Darmstadt, Germany). It was dissolved in dimethyl sulfoxide (DMSO) (Merck, Darmstadt, Germany) and then diluted in medium.

Vitamin K2 + Chitosan Solution

Five mL of the prepared vitamin K2 solution was added to 5 mL of chitosan solution. The solutions were mixed well to obtain a homogeneous mixture of vitamin K2 and chitosan.

nHAP + Chitosan Solution

Five mL of chitosan solution was combined with nHAP, and the mixture was extensively stirred to achieve consistent distribution of nano-hydroxyapatite within the chitosan matrix.

VK2 + Chito + nHAP Solution

The vitamin K2 + chitosan solution (5 mL) was combined with the nHAP, and the solutions were mixed well to create the VK2 + Chito + nHAP solution.

Microbial strains and inoculum preparation

The microorganisms utilized in this research were procured from the Culture Collections of the Nanobiomedicine Laboratory at Saveetha Dental College in Chennai. The selected strains included *Staphylococcus aureus* (*S. aureus*), *Streptococcus mutans* (*S. mutans*), *Enterococcus faecalis* (*E. faecalis*), and *Candida albicans* (*C. albicans*). To facilitate the growth and assessment of these microorganisms, 100 mL of Mueller Hinton agar (HiMedia Labs, India) was meticulously prepared for *S. mutans*, *S. aureus*, and *E. faecalis*, while Rose Bengal agar (HiMedia Labs, India) was specifically formulated for *C. albicans*. Following preparation, the agar underwent sterilization and was subsequently poured into Petri plates, allowing it to solidify. The bacterial and fungal culture plates were then subjected to incubation at optimal growth temperatures, with bacterial cultures being observed over a 24-hour period and fungal cultures for 48 hours, providing an appropriate environment for the assessment of antimicrobial activities.

Agar well diffusion test

The antimicrobial effectiveness of the test substances was evaluated employing the agar well diffusion method. In his approach, a 50 μL volume of the respective compound test solution was uniformly distributed on the agar plates to establish a consistent microbial lawn. The inoculation involved three even streaks across the entire plate surface, with the Petri plates rotated at approximately 60° between each application, followed by swabbing around the periphery of the agar surface. Subsequently, four wells, each measuring 7 mm in diameter and 4 mm in depth, were strategically positioned at equal distances on the plate. A 70 μL volume of each test compound solution was dispensed into four of the wells using micropipettes. The plates were then incubated at 37°C for 24 hours aerobically for *S. mutans*, *S. aureus*, *E. faecalis* in Sabouraud Dextrose Agar (SDA) and *C. albicans* in Rose Bengal agar. After the incubation period, the Petri plates were examined for the presence of zones of inhibition, which were measured using a millimeter scale. To ensure reliability, the entire protocol was repeated three times to minimize experimental errors.

Zone of inhibition measurement

Following the incubation, the size of the inhibition zones surrounding the impregnated disks was assessed by placing a ruler against the rear of the petri plate. The dimensions of the restrained growth zones were gauged, rounded to the closest whole millimeter. Zones are indicative of the antimicrobial activity of the test compounds against the tested microbial strains. 

Statistical analysis

Descriptive statistics was done to assess the mean and standard deviation. The mean zone of inhibition and standard deviation (SD) were measured as indicators of the antimicrobial activity, with a standard reference for comparison. To compare the mean between groups, one-way ANOVA was used ,whereas to compare the concentration intervals, post hoc test was used. To analyze the data, IBM SPSS Statistics for Windows, 26.0 (Released 2019; IBM Corp., Armonk, New York, United States) was used. P-value < 0.05 was considered to be statistically significant.

## Results

The overall mean zone of inhibition among the study groups at different concentrations is given in Table 2. Significant differences in the mean concentrations were observed among the study groups and dosages. Notably, in the K2 + Chito + nHAP group, the dosage of 25 µL exhibited a significantly higher mean concentration compared to the standard group (P = 0.027). Similarly, the vitamin K2 + chitosan group at 25 µL dosage displayed a significantly higher mean concentration compared to the standard group (P = 0.040).

**Table 2:** Comparison of the Overall Mean Zone of Inhibition Among the Study Groups at Different Concentrations nHAP: Hydroxyapatite nanoparticles; Chito: chitosan.

Groups	Concentration	Mean	SD	P-value
Vitamin k2 + nHAP	25 µL	11.5	2.64	0.161
	50 µL	15.0	5.16	
	100 µL	17.5	7.14	
	Standard	10.2	0.95	
K2 + Chito + nHAP	25 µL	12.5	3.69	0.027*
	50 µL	17.2	6.18	
	100 µL	21.5	6.60	
	Standard	10.0	0.0	
Vitamin K2	25 µL	9.0	0.0	0.58
	50 µL	9.0	0.0	
	100 µL	9.5	1.0	
	Standard	9.5	1.0	
Vitamin K2 + Chitosan	25 µL	10.2	1.25	0.040*
	50 µL	13.0	4.20	
	100 µL	18.0	6.97	
	Standard	9.0	0.0	

Significant differences in the intergroup comparison were identified in the mean values across various study groups and dosages. Specifically, in the comparison between the K2 + Chito + nHAP (100 µL) group and the vitamin k2 + nHAP (standard) group, a substantial difference was observed, with the former exhibiting a significantly lower mean value (p = 0.019). Similarly, in the comparison between the K2 + Chito + nHAP (100 µL) group and the vitamin K2 + nHAP (25 µL) group, a notable difference emerged, indicating a lower mean value in the former (p = 0.006). Furthermore, the vitamin K2 + nHAP (standard) group demonstrated a significantly lower mean than the K2 + Chito + nHAP (100 µL) group (p = 0.006). These findings underscore the importance of considering the dosage effects when interpreting the impact of different formulations on the variable of interest.

**Figure 1:**
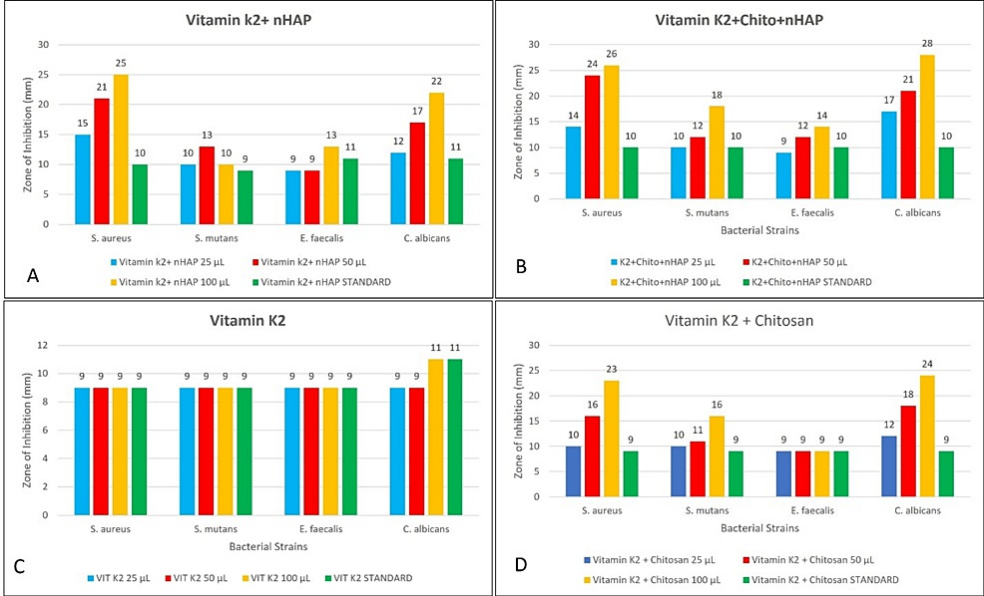
Graphical illustration of Antimicrobial Effectiveness at Various Concentrations of Different Compounds Antimicrobial activity of all the groups (A: vitamin K2 + nHAP; B: vitamin K2 + Chito + nHAP; C: vitamin K2; D: vitamin K2 + chitosan) at varying concentrations. The X axis represents the concentrations of the respective test compounds at 25 µL, 50 µL, and 100 µL, while the Y axis represents the zone of inhibition in mm.

In Figure 1A, the antimicrobial activity of vitamin K2 + nHAP is evident, with concentration-dependent increases in the inhibition zones against *S. aureus*, *S. mutans*, *E. faecalis*, and *C. albicans*. At a 100 µL concentration, the compound showed significant effectiveness, achieving zones of inhibition of 25 mm for *S. aureus*, 13 mm for *S. mutans*, 13 mm for *E. faecalis*, and 22 mm for *C. albicans*. Figure 1B illustrates a similar trend for K2 + Chito + nHAP, with *C. albicans *displaying the highest sensitivity, reaching a 28 mm inhibition zone at a 100 µL concentration. *S. aureus *and *S. mutans* also exhibited notable sensitivity across different concentrations, while *E. faecalis *showed moderate sensitivity, with an 18 mm inhibition zone at 100 µL concentration.

Figure 1C demonstrates the antimicrobial activity of vitamin K2 alone, revealing a consistent but modest zone of inhibition (9 mm) across all concentrations. The inhibitory effects were comparatively weaker against *S. aureus*, *S. mutans*, and *E. faecalis*, while a slightly increased sensitivity was observed against *C. albicans* at a 100 µL concentration, with an 11 mm inhibition zone. In Figure 1D, the combination of vitamin K2 and chitosan displayed significant antimicrobial activity. The inhibition zones increased with rising concentrations, with *C. albicans* exhibiting the highest sensitivity (24 mm at 100 µL). *S. aureus* and *S. mutans *also showed considerable sensitivity at varying concentrations, while *E. faecalis *displayed moderate sensitivity, with a 16 mm inhibition zone at a 100 µL concentration.

## Discussion

This study aimed to explore the antimicrobial capabilities of the nanocomposite composed of nano-hydroxyapatite, chitosan, and vitamin K2 against a range of microorganisms with clinical significance.

The integration of bioactive compounds into medical implant coatings offers significant potential in the fight against antimicrobial drug resistance. The risk of peri-implantitis, a common complication associated with implants, can be minimized by combining antimicrobial agents with implant coatings since they can act as a protective barrier which prevents biofilm formation. This approach of incorporating antimicrobial compounds alongside bioactive substances provides an innovative pathway to develop versatile materials with enhanced antimicrobial properties and added therapeutic benefits.

The investigation assessed the antimicrobial activity of vitamin K2 + nHAP, K2 + chitosan + nHAP, vitamin K2, and vitamin K2 + chitosan. The results demonstrated differential antimicrobial efficacy among the tested compounds against *S. aureus*, *S. mutans*, *E. faecalis*, and *C. albicans* since these microbial strains are responsible for peri-implantitis and subsequent implant failures [14]. The differential responses of the microbial strains to the nanocomposite may be attributed to variations in their cell wall structures and susceptibilities to the antimicrobial agents.

Among the tested compounds, vitamin K2 + nHAP exhibited a concentration-dependent increase in zones of inhibition indicating that higher concentrations of vitamin K2 + nHAP had more pronounced inhibitory effects against the microorganisms. The observed variation in the zones of inhibition for the different microbial strains could be attributed to differences in their susceptibility to the formulation. *S. aureus *and *C. albicans* appear to be more sensitive to the compound, as evidenced by larger zones of inhibition at all concentrations. In contrast, *S. mutans* and* E. faecalis* show slightly reduced sensitivity, with smaller zones of inhibition at certain concentrations. The observed antimicrobial activity of vitamin K2 + nHAP nanocomposite can be attributed to the synergistic interplay between vitamin K2 and nHAP. Vitamin K2 is acknowledged for its inherent antimicrobial properties, capable of disrupting microbial cell membranes and interfering with crucial cellular processes. Concurrently, nHAP, as a biocompatible material, acts synergistically to augment the overall efficacy of the composite. It creates a conducive environment that facilitates sustained release of the antimicrobial agent, thereby enhancing its bioavailability and prolonging its activity. The combined action of vitamin K2 and nHAP thus results in a potentiated antimicrobial effect, where their individual strengths complement each other, leading to improved performance against a range of microbial strains [15].

Vitamin K2, also known as menaquinone, is an aromatic fat-soluble molecule. When combined with other bioactive compounds, their sustainable release can be due to hydrophobic interactions between the drug compound [16]. This interaction leads to the aggregation of the compound. This led to an accelerated diffusion process from the surface of the implant, resulting in improved release kinetics that makes vitamin K2. Menaquinone is considered as a plausible candidate for the formulation of efficacious antibacterial agents with minimal untoward effects, since it is exclusively present in anaerobic and Gram-positive bacteria. The recent understanding of menaquinone biosynthesis and its critical roles in microbial growth has identified potential antimicrobial agents, with many inhibitors resembling the substrate or cofactors of the biosynthetic enzymes. Leveraging the unique properties of vitamin K2 offers a promising avenue for developing innovative antimicrobial strategies to combat drug-resistant microorganisms effectively. By combining their individual strengths, these compounds may provide a synergistic approach to address the challenges of antimicrobial resistance and improve the efficacy of antimicrobial therapies [10,17,18]. 

**Figure 2:**
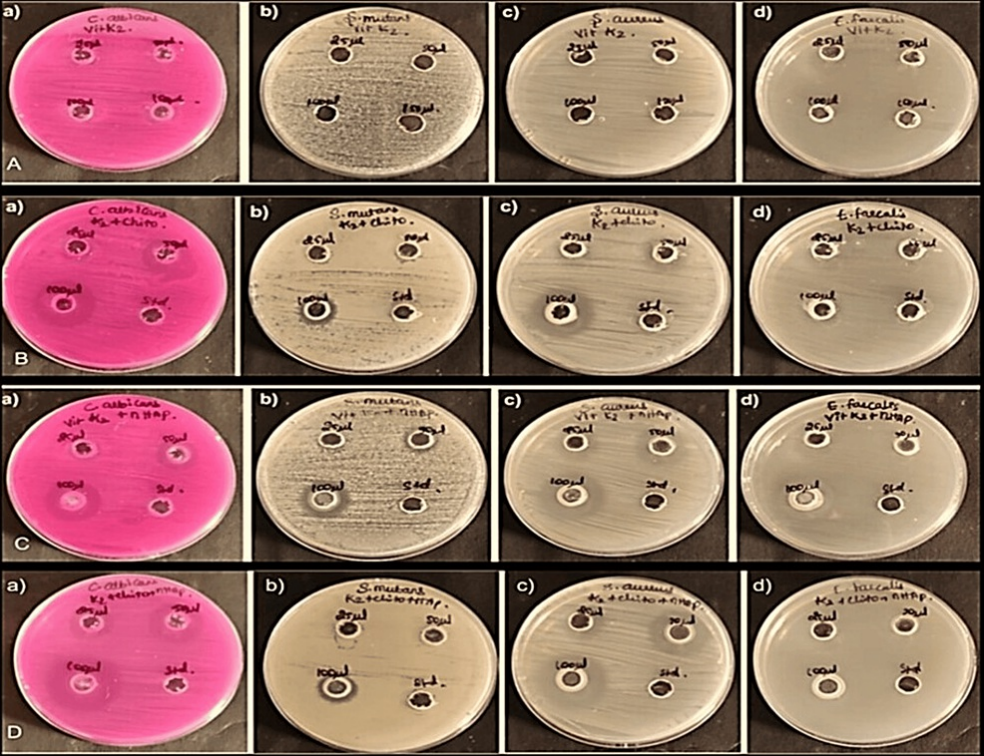
Antimicrobial Activity of Vitamin K2 (A), Vitamin K2 and Chitosan (B), Vitamin K2 and nHAP (C), and Vitamin K2, Chitosan, + nHAP (D) [(a) C. albicans, (b) S. mutans, (c) S. aureus, (d) E. faecalis] 2A: Antimicrobial activity of vitamin K2 a) C. albicans, b) S. mutans, c) S. aureus, d) E. faecalis. 2B: Antimicrobial activity of vitamin K2 and chitosan a) C. albicans, b) S. mutans, c) S. aureus, d) E. faecalis. 2C: Antimicrobial activity of vitamin K2 and nHAP a) C. albicans, b) S. mutans, c) S. aureus, d) E. faecalis. 2D: Antimicrobial activity of vitamin K2, chitosan, and nHAP a) C. albicans, b) S. mutans, c) S. aureus, d) E. faecalis.

In our study [Figure 2], K2 + chitosan + nHAP exhibits significant antimicrobial activity against the tested microbial strains. Similar to the vitamin K2 + nHAP nanocomposite, the K2 + Chito + nHAP nanocomposite also demonstrated a dose-dependent increase in the zones of inhibition, indicating enhanced inhibitory effects at higher concentrations. At 50 µL, the zones of inhibition expanded to 24 mm, 12 mm, 12 mm, and 21 mm for *S. aureus*, *S. mutans*, *E. faecalis*, and *C. albicans*, respectively. *S. aureus* and *S. mutans* exhibited moderate sensitivity to the nanocomposite, since larger zones of inhibition was observed at higher concentrations. *E. faecalis* displayed moderate sensitivity, and its zone of inhibition increased with an increase in nanocomposite concentration. *C. albicans* showed significant sensitivity to the nanocomposite, with notably larger zones of inhibition at all concentrations tested. The larger zones of inhibition compared to the standard control indicate that this nanocomposite has the potential to combat microbial growth more effectively. The highest sensitivity was noted against *C. albicans* and *S. aureus* indicating its potential as an effective antimicrobial agent against these pathogens. However, moderate inhibitory effects against *S. aureus* and *S. mutans* indicate the need for further optimization of the formulation to improve its efficacy against Gram-positive bacteria. Similarly, the K2 + Chito + nHAP nanocomposite displayed potent antimicrobial activity against all tested microorganisms. The significant zones of inhibition observed at different concentrations indicate its broad-spectrum efficacy.

*C. albicans* displayed remarkable sensitivity, indicating a potential application of this nanocomposite in antifungal therapies. This aligns with previous findings, where chitosan-nHAP composite layers demonstrated inhibitory effects on fungal biofilm development. This inhibition is attributed to the interaction between the positively charged surface of the implant materials and the anionic components from the microbial strain structure, as observed in studies referencing such interactions, including those involving lipopolysaccharides (LPSs) [19].

When vitamin K was added to the chitosan-nHAP composite, it was found to further enhance the beneficial properties of the material. Vitamin K promotes bone health and facilitates proper bone mineralization [20,21]. Incorporation of vitamin K into the composite can contribute to the overall bone regeneration potential of the material, complementing the osteoconductive properties of hydroxyapatite. The antimicrobial activity of vitamin K, when combined with the existing antimicrobial properties of the chitosan-nHAP composite, can create a synergistic effect, leading to a more effective inhibition of microbial growth and biofilm formation. The inclusion of vitamin K could also play a role in modulating immune responses [22], which may further support tissue integration and reduce the risk of implant-related complications.

In contrast, pure vitamin K2 exhibited limited antimicrobial activity against the tested microorganisms. The consistent small zone of inhibition observed across all concentrations suggests weak inhibitory effects. While the compound displayed slightly increased sensitivity against *C. albicans* at higher concentrations, it lacked significant activity against the bacterial strains. The findings suggest that vitamin K2 might not possess sufficient standalone antimicrobial efficacy. However, its potential as an antimicrobial agent could be enhanced when synergistically combined with nHAP or chitosan.

K2 + Chito + nHAP and the vitamin K2 + chitosan in this study demonstrated moderate to significant sensitivity against *E. faecalis*, with zone sizes of 18 mm and 16 mm, respectively, at a concentration of 100 µL. The results of the study were in agreement with Wang et al. where it was observed that chitosan had a better antibacterial effect on *E. faecalis* since the bacteria proliferates in nonculturable conditions, as evidenced by its ability to maintain viability for an impressive one-year period even without a nutrient source [23]. The ability of microorganisms to survive is ascribed to the development of biofilms, which play a pivotal role as vital environmental adaptations. These biofilms act as protective and supportive mechanisms, aiding microorganisms in withstanding adverse conditions and antibiotic treatments [24]. Biofilms play a significant role in providing a shield that allows *E. faecalis* to withstand adverse environments, ensuring higher internal environment stability and prolonged viability [24].

In the context of combating *E. faecalis* and its biofilm-mediated survival, our investigation evaluated the antimicrobial activity of various test compounds. Among them, the K2 + Chito + nHAP nanocomposite and the vitamin K2 + chitosan combination emerged as the most promising candidates, displaying moderate to significant sensitivity against *E. faecalis*. At a concentration of 100 µL, these compounds demonstrated impressive zone sizes of 18 mm and 16 mm, respectively, inhibiting *E. faecalis *growth. Conversely, pure vitamin K2 exhibited weaker antimicrobial activity against *E. faecalis*, with a smaller inhibition zone of 9 mm at the same dosage. The vitamin K2 + nHAP nanocomposite showed moderate sensitivity, with an inhibition zone of 13 mm at 100 µL. These findings highlight the potential of the K2 + Chito + nHAP nanocomposite and the vitamin K2 + chitosan combination as effective agents to combat *E. faecalis* and its biofilm-associated survival.

The results collectively highlight the synergistic effects of combining vitamin K2 with nHAP or chitosan, leading to enhanced antimicrobial activity. The nanocomposite formulations appear to offer advantages over pure vitamin K2 in terms of broader spectrum efficacy and increased potency. The presence of nHAP and chitosan seems to contribute significantly to the improved antimicrobial properties, as evidenced by the larger zones of inhibition observed with the nanocomposites. The structural differences in the cell walls of these bacteria contribute to distinct susceptibilities to chitosan. Gram-negative bacteria, characterized by the presence of LPS and negatively charged surfaces, are thought to be more susceptible to chitosan due to electrostatic interactions. Chitosan, being cationic, can bind to the negatively charged phospholipids when the environmental pH is below 6.5. However, the antimicrobial action of chitosan is not solely reliant on electrostatic interactions, as evidenced by the increased resistance of *S. aureus* with the deletion of teichoic acid biosynthesis, suggesting a more complex mode of action. Gram-positive bacteria, with thicker peptidoglycans and negatively charged teichoic acids, also exhibit susceptibility to chitosan. Despite the thick cell wall in Gram-positive bacteria, certain chitosan oligomers can penetrate the cell wall, influencing processes such as DNA/RNA or protein synthesis. The molecular size of chitosan plays a crucial role in its antimicrobial activity, with low-molecular-weight chitosan and oligo-chitosan potentially affecting DNA/RNA or protein synthesis after passing through the cell wall and cell membrane into the cytoplasm. Chitosan's antimicrobial activity is further attributed to its ability to chelate environmental ions and nutrients required for bacterial survival [25-27].

This study marks the first of its kind to explore the synergistic effects of combining vitamin K2 with nHAP and chitosan for enhanced antimicrobial activity. These nanocomposites could find applications in wound dressings, biomedical coatings, and implant materials, to effectively combat infections and reduce the incidence of healthcare-associated infections [28]. However, further investigations are necessary to elucidate the underlying mechanisms responsible for the observed antimicrobial effects and to assess the biocompatibility and safety of the nanocomposites for clinical use.

Limitations

While our study reveals promising antimicrobial potential in nanocomposites containing vitamin K2, nHAP, and chitosan, several limitations should be acknowledged. The investigation primarily relies on in vitro assessments, lacking the complexity of in vivo conditions, and the selected microbial strains may not fully represent the clinical diversity encountered. The qualitative nature of the disk diffusion test offers valuable insights but falls short of providing quantitative data on antimicrobial efficacy. Additionally, the study lacks in vivo experiments, hindering a comprehensive understanding of the nanocomposites' real-world applicability and potential side effects. Cytotoxicity assessments, optimization of component ratios, and exploration of nanocomposite stability over time are also essential aspects not addressed in this study. Further research and clinical trials are imperative to validate the practical clinical relevance and safety and optimize the efficacy of these nanocomposites for potential biomedical applications.

## Conclusions

Vitamin K2 + Chito + nHAP and the vitamin K2 + chitosan combination demonstrated heightened sensitivity against clinically relevant microbial strains. These nanocomposites hold significant potential as effective antimicrobial agents to combat antibiotic-resistant microbes and improve infection control in biomedical applications. When it is coated on implant surfaces as coating material, these nanocomposites will enhance osseointegration in patients with low bone quality, for example, women with menopause, hypoparathyroidism, and low vitamin D level.
